# A Pediatric Case of Granulomatous Myositis and Response to Treatment

**DOI:** 10.7759/cureus.14507

**Published:** 2021-04-15

**Authors:** Rabheh Abdul-Aziz, Henry J Sioufi, Chrisana Pokorny, Rabi Tawil

**Affiliations:** 1 Pediatric Rheumatology, Wolfson Children's Hospital, Jacksonville, USA; 2 Pediatrics, University at Buffalo, Buffalo, USA; 3 Neurology, Strong Memorial Hospital, Rochester, USA

**Keywords:** idiopathic inflammatory myopathy, granulomatous myositis, rituximab

## Abstract

Idiopathic inflammatory myopathy encompasses a group of acquired, heterogeneous, systemic diseases of the skeletal muscle, including adult polymyositis, adult dermatomyositis, juvenile dermatomyositis, juvenile polymyositis, inclusion body myositis, and necrotizing myopathy, all resulting in muscle weakness. Granulomatous myositis (GM) is a rare myopathy disorder histologically characterized by the development of endomyseal and/or perimyseal granulomas in striated muscle. GM is often associated with sarcoidosis. GM has also been associated with myasthenia gravis, inflammatory bowel disease, thymoma, and malignancy. We are reporting a rare case of a 13-year-old girl with GM without associated disease that was refractory to multiple medications, and responded well to rituximab.

## Introduction

Idiopathic inflammatory myopathy encompasses a group of acquired, heterogeneous, systemic diseases of the skeletal muscle, including adult polymyositis, adult dermatomyositis, juvenile dermatomyositis, juvenile polymyositis, inclusion body myositis, and necrotizing myopathy, all resulting in muscle weakness [1]. Major symptoms of myositis include pain and weakness of the arms and legs causing difficulty in walking, climbing stairs, and lifting objects above the head [2]. Dermatomyositis affects both adults and children, while other forms of myositis are more common in middle-aged individuals [3]. Granulomatous myositis (GM) is a rare neuromuscular disorder histologically characterized by the development of endomyseal and/or perimyseal granulomas in striated muscle. Clinical hallmarks of the disease include proximal and distal muscle weakness, myalgia, and bulbar symptoms [4]. GM is often associated with sarcoidosis, myasthenia gravis, inflammatory bowel disease, thymoma, and malignancy [5,6].

We are reporting a rare case of a 13-year-old girl with GM that was refractory to multiple medications, and responded well to rituximab.

## Case presentation

Patient is a 13-year-old Caucasian female with GM diagnosed in 2016 at the age of 9 years old, confirmed with muscle histopathology. Prior to presentation, she was seen by her primary care physician for walking without bending her knee; a problem that has persisted since the age of 2. She also noted that she became fatigued with associated muscle weakness resulting in the inability to keep up with her peers. She was seen by neurology with normal electromyography (EMG) and subsequent muscle biopsy of the right quadriceps showed granuloma concerning for sarcoidosis and she was referred to rheumatology.

**Figure 1:**
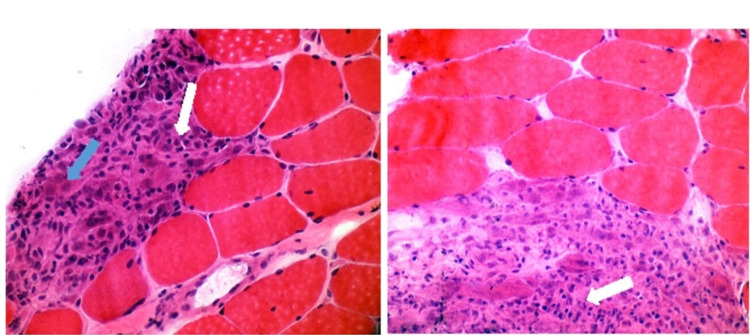
Muscle biopsy. Hematoxylin and eosin staining showing two multiple small non-caseating granulomas with mononuclear inflammatory cells with dark nuclei and minimal cytoplasm, typically at the periphery of the granulomas (white arrows) and histiocytes with large pale nuclei and abundant cytoplasm (blue arrows), the precursors of multinucleated giant cells.

She was seen at our Rheumatology clinic, where her physical exam showed right knee swelling without muscle weakness, and her childhood myositis assessment score (CMAS) was 52/52. Exam did not reveal any rashes or abnormal nailfold capillaries. Blood work showed elevated CK 4750 U/L (normal <170 U/L), elevated aldolase 40 U/L (normal <10 U/L), elevated AST 120 U/L (normal <50 U/L), elevated ALT 212 U/L (normal <50) and normal Lactate dehydrogenase. Work up for sarcoidosis showed unremarkable chest CT with no evidence of nodule or lymphadenopathy. Eye exam was unremarkable. Lysozyme, angiotensin-converting enzyme (ACE), and urine calcium all normal. NOD2 gene mutations were negative, making Blau Syndrome unlikely. Chest CT scan showed normal thymus size making thymoma with myasthenia gravis less likely. Acetylcholine receptor antibody was negative. Blood work also showed normal Neopterin, normal Von Willebrand, negative antineutrophil cytoplasmic antibodies (ANCA), negative antinuclear antibodies (ANA), negative rheumatic factor, negative mitochondria antibody, negative Jo 1 antibodies, and negative smooth muscle antibodies. Myositis antibodies panel was denied by her insurance. She has normal thyroid function, normal immunoglobulins, normal ceruloplasmin and copper level. She had a negative workup for tuberculosis, hepatitis A, B and C, cytomegalovirus, and Epstein-Barr virus. Review of her muscle biopsy of the right quadriceps muscle showed increased variability in fiber size due to the presence of atrophy. One cluster of necrotic fibers was seen, all of which were undergoing phagocytosis. There were multiple perimysial foci of inflammatory cells, often centered around blood vessels. These foci had few mononuclear cells but many epithelioid cells, some with multiple nuclei suggestive of granulomas. Step sections of the frozen tissue showed additional granulomatosis foci as well as one area with endomysial mononuclear inflammation and multiple necrotic fibers (Figure 1).

**Figure 2:**
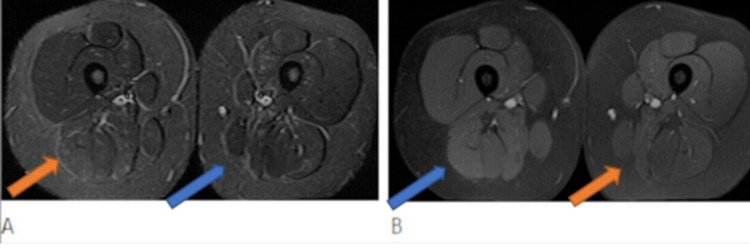
MR images of the lower thighs. Axial MR images of the lower thighs. A: STIR image demonstrates hyperintense signal in the muscles of posterior compartment. B: Post contrast T1 weighted image demonstrates subtle enhancement within the areas of signal abnormality of the muscles.

She was evaluated by Gastroenterology. Esophagogastroduodenoscopy and colonoscopy showed no evidence of Crohn’s disease. She was started on weekly intravenous Methylprednisolone and subcutaneous Methotrexate. Months later, the patient self-discontinued her Methotrexate and symptoms of muscle weakness persisted. Muscle MRI of the bilateral lower extremities showed mild inflammatory changes in the long head of the rectus femoris muscles, right more than left and mild inflammatory changes within the semimembranous muscles, left more than right (Figure 2). She restarted oral methotrexate and infliximab 5 mg/kg, monthly infusions were established after initial induction therapy. She had some improvement with decreased AST from 100 to 75 U/L and decreased CK from 5390U/L to 2919 U/L. ALT remained elevated at 159 U/L. Clinically, she was without muscle weakness with CMAS 52/52, but reported occasional slowness with physical activity.

During her follow-up in May 2018, she continued to do well clinically, CMAS 52/52, but was noted to have a significant elevation in muscle enzymes with CK 12,159, aldolase 111, AST 207, and AST 240. She was started on oral Prednisone 1 mg/kg daily with tapering. Repeated blood tests showed elevated CK 3840, elevated aldolase 60, elevated AST 101, elevated ALT 161, and elevated LDH 391. She continued on oral prednisone with tapering and oral methotrexate, infliximab was discontinued. While decreasing her steroids, blood work showed elevated aldolase 165, elevated ALT 247, elevated AST 169, elevated LDH 650, and significantly elevated CK 18038. Muscle enzymes continued to be elevated despite increasing her dose of daily prednisone. She once again self-discontinued methotrexate. Physical exam showed CMAS 49/52 with inability to hold her arms, legs, and head up for 2 minutes; she elicited significant pain in her arms and legs after CMAS. Since she was experiencing significant weight gain from the high-dose prednisone without improvement in her muscle enzymes, the decision was made to wean the steroids and start IVIG.

IVIG infusions started in October of 2018. Clinically, she continued to have weakness with competitive activities. Her muscle enzymes improved: CK 5042, AST 105, ALT 148, LDH 361, aldolase 41. After four months of IVIG therapy, no additional improvement was noted and her blood work showed CK 5864, AST 150, ALT 138, LDH 351, aldolase 45. The decision to start rituximab was discussed with the family if she continued to show no improvement on IVIG.

She presented for follow-up in May 2019. She had discontinued all treatment at this time as symptoms had improved with only occasional muscle pain or weakness noted with activity. CMAS was 52/52. Muscle enzymes continued to be elevated with CK 2439, AST 48, ALT 83, LDH 287, aldolase 18.2. Muscle enzymes have remained persistently elevated despite Methotrexate, high dose steroids, infliximab, and IVIG. Due to the refractory nature of her myositis and the long-term risk of irreversible muscular fibrosis, her family agreed to begin Rituximab infusions.

Patient was once again lost to follow up until January 2020. Her blood work showed CK 2543, AST 65, ALT 82, LDH 235, aldolase 30. Per parent’s request, CPT2 testing was normal. Her parents were resistant to additional therapy as they felt she was asymptomatic with no muscle weakness.

**Figure 3:**
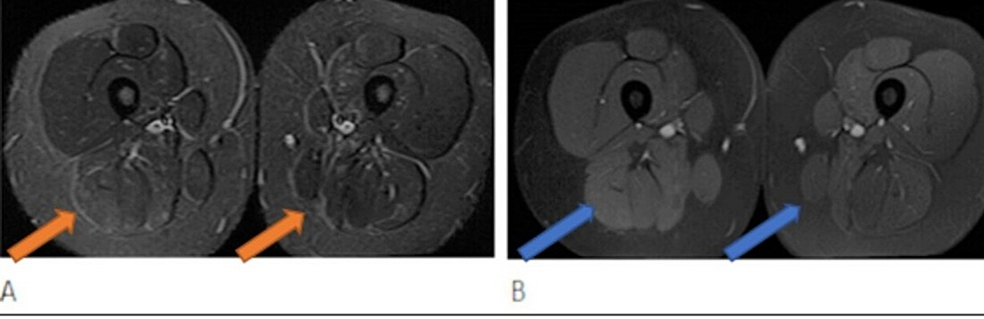
MR images of the lower thighs. Axial MR images of the lower thighs in 2020. A: STIR image demonstrates hyperintense signal in the muscles of posterior compartment, like the study from 2017. B: Post contrast T1 weighted image demonstrates subtle enhancement within the areas of signal abnormality of the muscles, like the prior study from 2017 (see Figure 2).

Blood work in August of 2020 showed CK 2688, AST 58, ALT 58, LDH 246, aldolase 25. CD19 count normal. Bilateral lower extremity MRI in September 2020 demonstrated previously identified edema within the long head of the biceps femoris muscle bilaterally right greater than left that persists with mild patchy enhancement within these muscles right greater than left (Figure 3).

After discussion with the family again, rituximab infusion was started in November 2020. She received two doses of 750 mg/m^2^ on day 0 and 15. Prior to initial dose, CMAS 52/52, CK 4657, AST 113, ALT 116, LDH 285, aldolase 40.8. Significant improvement was seen following rituximab with CMAS 52/52, CK 952, AST 36, ALT 49, LDH 191, and aldolase 12; these are the lowest recorded values for muscle enzymes since presentation to clinic.

## Discussion

Granulomatous myositis is a myopathic syndrome associated with non-specific epithelioid granulomas in striated muscle [2,4,7,8]. This rare entity is most frequently related to sarcoidosis, but other uncommon causes have been reported [4]. Idiopathic GM is the diagnosis after systemic disorders known to cause similar myopathological abnormalities have been excluded [4,5]. Symmetrical proximal or distal muscle weakness is the rule in the clinical presentation. Although the clinical profile together with EMG studies may be useful, definite diagnosis requires pathological examination [4].

Muscle MRI can be used as a guide for a muscle biopsy given the patchy pattern of involvement [9]. This patient was diagnosed with granulomatous myositis confirmed with histopathology and extensive workup to rule out inflammatory bowel disease, myasthenia gravis, thymoma, sarcoidosis, and Blau’s syndrome.

Early diagnosis and treatment of myositis are crucial for long-term outcome and delay can lead to irreversible muscle damage with fibrosis, muscle atrophy, or fat replacement [2]. Systemic glucocorticoids are the treatment of choice, but the clinical outcome is not always satisfactory [4]. Idiopathic GM is a rare entity reported in adult medicine, and is even more rare in pediatrics. For this reason, there are no guidelines for treatment in the pediatric population [10,11]. This patient has disease that was refractory to high dose steroids, methotrexate, infliximab, and IVIG with persistent elevation in muscle enzymes and evidence of inflammation on MRI.

Rituximab has been used in refractory cases of adult polymyositis and both adult and pediatric dermatomyositis with a recent literature review showing a 78% response rate to the medication [8]. Subsequently, rituximab was discussed in this case and so far, she has had the best response on this therapy in comparison to the various medications that have been tried over the past five years. She will need future follow-up to evaluate sustained response.

## Conclusions

We reported a rare case of a 13-year-old girl with GM. Disease course was very rocky and refractory to multiple medications but responded well to rituximab.
